# How often does white matter hyperintensity volume regress in cerebral small vessel disease?

**DOI:** 10.1177/17474930231169132

**Published:** 2023-05-09

**Authors:** Robin B Brown, Daniel J Tozer, Marco Egle, Anil M Tuladhar, Frank-Erik de Leeuw, Hugh S Markus

**Affiliations:** 1 Department of Clinical Neurosciences, University of Cambridge, Cambridge, UK; 2 Department of Neurology, Centre for Cognitive Neuroscience, Donders Institute for Brain, Cognition and Behaviour, Radboud University Medical Centre, Nijmegen, The Netherlands

**Keywords:** Brain, leukoaraiosis, lesions, magnetic resonance imaging, cerebral infarction, ischemic stroke

## Abstract

**Background and objectives:**

It has been suggested that white matter hyperintensity lesions (WMHs), which typically progress over time, can also regress, and that this might be associated with favorable cognitive performance. We determined the prevalence of WMH regression in patients with cerebral small vessel disease (SVD) and examined which demographic, clinical, and radiological markers were associated with this regression.

**Methods:**

We used semi-automated lesion marking methods to quantify WMH volume at multiple timepoints in three cohorts with symptomatic SVD; two with moderate-to-severe symptomatic SVD (the SCANS observational cohort and the control arm of the PRESERVE interventional trial) and one with mild-to-moderate SVD (the RUN DMC observational cohort). Mixed-effects ordered logistic regression models were used to test which factors predicted participants to show WMH regression.

**Results:**

No participants (0/98) in SCANS, 6/42 (14.3%) participants in PRESERVE, and 6/276 (2.2%) in RUN DMC showed WMH regression. On multivariate analysis, only lower WMH volume (OR: 0.36, 95% CI: 0.23–0.56) and better white matter microstructural integrity assessed by fractional anisotropy using diffusion tensor imaging (OR: 1.55, 95% CI: 1.07–2.24) predicted participant classification as regressor versus stable or progressor.

**Discussion:**

Only a small proportion of participants demonstrated WMH regression across the three cohorts, when a blinded standardized assessment method was used. Subjects who showed regression had less severe imaging markers of disease at baseline. Our results show that lesion regression is uncommon in SVD and unlikely to be a major factor affecting the use of WMH quantification as an outcome for clinical trials.

## Introduction

Cerebral small vessel disease (SVD) is the most common cause of vascular cognitive impairment and causes approximately 25% of ischemic strokes.^1^ Most cases are sporadic and typically associated with cardiovascular risk factors;^2^ however, despite its importance, understanding of the underlying pathophysiology is incomplete and there are few effective disease-modifying treatments.^3^

White matter hyperintensities (WMHs) are a radiological hallmark of SVD and independently predict both stroke and dementia.^4^ These areas of presumed chronic ischemia are best seen on T2-weighted magnetic resonance imaging (MRI) as high signal in subcortical and brainstem structures. Observational studies in community-dwelling participants have shown that the total volume of such lesions increases over time.^5–7^ WMH progression has also been assessed in specific patient groups with ischemic stroke,^8^ hypertension,^9^ and unselected cardiovascular disease^10,11^ in which longitudinal lesion growth has been demonstrated. WMHs are readily quantifiable and increasingly used as an outcome measure (particularly in SVD trials) to test interventions in smaller sample sizes prior to large clinical trials with the endpoints of stroke and dementia.^12–14^

Although WMH volume tends to increase over time, there have been reports of WMH regression in a proportion of patients with stroke and SVD,^15,16^ with regression noted in up to 37% of participants in a population with minor stroke.^17^ A recent meta-analysis found that WMH regression was documented explicitly in one-third of MRI studies investigating adults with sporadic SVD.^18^ This finding has potentially important implications. If WMHs do regress, then better understanding of this process may inform potential treatment approaches. Second, it adds complexity to power calculations required for trial design and might increase requisite sample sizes for interventional studies. Various reasons have been suggested for observed WMH regression, including radiological and analytical factors.^19^

To determine the frequency of WMH lesion volume regression while accounting for possible technical factors, we examined three cohorts of patients all with symptomatic SVD: in two, this was defined as a clinical lacunar stroke syndrome with a corresponding lacunar infarct and confluent WMHs on MRI (St George’s Cognition and Neuroimaging Study (SCANS) and PRESERVE) and in one, as any clinical symptoms compatible with and any radiological evidence of SVD on MRI (the Radboud University Nijmegen Diffusion Tensor and Magnetic Resonance Cohort (RUN DMC)). Using robust analysis techniques blinded to time point of scans, we determined whether regression of lesion load occurs across multiple time points and which patient and imaging factors (including more subtle measurements of white matter microstructural damage, such as diffusion tensor imaging (DTI)) are associated with this regression.

## Methods

### Study populations

Three cohorts of patients with symptomatic SVD and differing degrees of WMH severity were studied. All participants provided written, informed consent prior to enrollment.

The SCANS^20^ was a prospective observational study that recruited patients with symptomatically defined SVD presenting with a lacunar stroke syndrome and had both a compatible lacunar infarct and at least early confluent WMHs on MRI (Fazekas scale^21^ score ⩾ 2). MRI scans were performed at least 3 months post-stroke and annually for 3 years afterwards. Patients were recruited from three South London hospitals between March 2007 and October 2010, and the study was approved by the Wandsworth Research Ethics Committee (ukctg.nihr.ac.uk; study ID: 4577).

The PRESERVE study^13^ was a multicentre randomized control trial of intensive versus standard blood pressure treatment in SVD. Patients with a lacunar stroke syndrome (and a compatible lacunar infarct on MRI) and WMHs of Fazekas score ⩾ 2 were recruited at least 3 months post-stroke from six UK-wide hospitals between February 2012 and October 2015. MRI was performed at baseline and after 2 years; the trial was approved by the Harrow Research Ethics Committee (reference: 11/LO/0458) and registered with the International Standard Randomized Control Trial Number registry (reference: ISRCTN37694103). For the purposes of this study, we included only participants from the standard treatment arm because WMH change in the intensive arm may be confounded by the effects of intensive antihypertensive treatment.

The RUN DMC^22^ was a prospective long-term cohort study that recruited patients with any symptoms compatible with SVD and evidence of either lacune(s) or any WMHs on MRI. Patients were recruited at Radboud University Medical Center, Netherlands and had MRI scans in 2006, 2011, and 2015. Due to equipment upgrade after the first time point in the RUN DMC study, we used images from the second and third time point as baseline and follow-up time points. The study was approved by the Arnhem–Nijmegen Medical Research Ethics Committee (No. 2005/256).

### Image acquisition

The three cohorts included participants at least 3 months after stroke, at which point they are expected to be clinically stable^23^ and evidence of cavitation is likely to be visible already.^24^ MRI scanner and sequence details for the studies above have previously been published^20,22,25^ and are summarized here in brief and fully in the Supplemental material.

MRI imaging in SCANS was performed on a 1.5 Tesla GE Signa HD MRI scanner at St George’s, University of London and included T1-weighted, fluid-attenuated inversion recovery (FLAIR) and DTIs.

Images in PRESERVE were acquired using the following eight 3.0 Tesla MRI scanners across the six sites: Siemens Prisma, Siemens Magnetom Prisma Fit, Siemens Verio, Philips Ingenia, Philips Achieva, and three Philips Achieva TX. T1-weighted scans were acquired to produce 1 mm^3^ isotropic resolution, and repetition time (TR) and echo time (TE) were optimized to ensure T1-weighting/tissue contrast was comparable across sites. FLAIR sequences had identical inversion times and were also TE matched with TR long enough to minimize T1-weighting (representative TR/TE/TI 11,000/120/2800 ms, final resolution 0.48 × 0.48 × 3 mm). DTIs were acquired using 32 equally spaced non-collinear diffusion gradients at *b* = 1000 s/mm^2^ and eight unweighted images at *b* = 0 s/mm^2^ (representative *TR* = 6850 ms, *TE* = 75 ms; final resolution is 2.5 mm^3^ isotropic). T1-weighted, FLAIR, and DTI images were used for this analysis.

In the RUN DMC study, MRI was acquired using a 1.5 Tesla Siemens Magnetom Avanto and included T1-weighted, FLAIR, and DTI sequences.

### Image analysis

Two methods of measuring WMH lesion load were used. The analysis pipeline used in SCANS has previously been described.^26^ In brief, images were pre-processed using Statistical Parametric Mapping 8 (http://fil.ion.ucl.ac.uk/spm/software/spm8/) to check orientation and co-register to MNI registration in 1 mm isotropic voxels. Group average tissue probability maps were created using a modified multivariate mixture of Gaussians^27^ and individual images were then segmented using these custom maps and repaired manually if required using ITK-SNAP (http//www.itksnap.org) including the removal of any lacunes or hemorrhages. Each subject image was then warped to an individual participant midpoint average image to create divergence maps for voxels containing WMHs.

WMH lesion load was determined in PRESERVE and RUN DMC using Jim version 8.0 (http://xinapse.com/j-im-software/), a semi-automated program in which a region of interest is selected by the rater and voxels within this contour delineated. The program was run on a Microsoft^®^ Surface PC and manual adjustment performed using a stylus tool to correct lesion boundaries on the screen. To minimize errors relating to the selection of lesions, images were marked slice by slice on a parallel split screen and the image intensity was matched between scans. To reduce the risk of bias, images were randomly displayed in terms of order of acquisition and the rater was blinded to image time point. Any hemorrhages and isolated lacunes were not included in the lesion mask; in the case of lacunes within areas of confluent WMHs, the entire area of FLAIR high signal was marked and the area of low signal isointense with cerebrospinal fluid was then removed.

We used previously described criteria to determine whether there was progression or regression of WMH lesion load. Participants were categorized as “regressors” if they showed a decrease in the total WMH volume of at least 0.25 cubic centimeters (cc) between any sequential scans. This is the minimum difference between WMH volume that can be appreciated visually and has previously been used to define lesion regression.^28^ “Progressors” were defined as participants who showed an increase in greater than one standard deviation of the inter-scan change within each study as has also been used previously.^28^

DTI images in SCANS and PRESERVE were pre-processed using the Eddy correct tool from Functional MRI of the Brain’s Diffusion Toolbox (http://fsl.fmrib.ox.ac.uk/fsl/fslwiki/FDTref). In RUN DMC, the DTI images were pre-processed using an in-house iteratively reweighted least squares algorithm.^29^ In all three cohorts, fractional anisotropy (FA) and mean diffusivity (MD) histograms were created using the FMRIB’s DTIFit tool. White matter FA median and MD normalized peak height were used for analysis, as these DTI parameters have been shown to be reproducible between sites in SVD patients.^30^

Brain volumes in SCANS and RUN DMC were calculated by excluding the WMH lesion maps from the gray and white matter masks derived from SPM8, correcting for intracranial volume using SIENAX (https://fsl.fmrib.ox.ac.uk/fsl/fslwiki/SIENA) and adding the lesion masks. Brain volumes in PRESERVE were calculated using applying SIENAX to images that had been intensity non-uniformity corrected by N4ITK and segmented using SPM12 including correction for intracranial volume using SIENAX as above.

A comparison of the three studies, including population and imaging details, is given in Table 1.

**Table 1.** Comparison of the three included studies.

	SCANS	PRESERVE	RUN DMC
Population	Lacunar stroke + moderate-to-severe WMHs (Fazekas ⩾ 2)	Lacunar stroke + moderate-to-severe WMHs (Fazekas ⩾ 2)	Neurological symptoms + any signs of SVD on MRI
Time since most recent stroke (months, *M* ± *SD*)	34.8 ± 67.2	9.6 ± 17.6	
Study type	Observational cohort	Interventional (intensive blood pressure treatment vs control)	Observational cohort
Sites	Single center, UK	Multicentre, six UK sites	Single center, the Netherlands
No. of participants	99	84 (42 from control group used in this analysis)	276
MRI time points	Baseline 1 year 2 years 3 years	Baseline 2 years	Baseline 5 years 9 years
MRI scanner	1.5T GE Signa HD	Eight 3T scanners across sites	1.5T Siemens Magnetom Avanto
WMH analysis pipeline	Semi-automated: SPM8 using customized group average tissue probability maps warped between time points	Semi-automated: parallel time point-blind marking using contouring program (Jim v8.0)	Semi-automated: parallel time point-blind marking using contouring program (Jim v8.0)
Study period	2007–2010	2012–2015	2006–2015

Data from the SCANS and PRESERVE studies can be obtained from Hugh Markus on reasonable request. Data from RUN DMC can be obtained from Frank-Erik de Leeuw on reasonable request.

### Statistical analysis

Between-category differences in demographic factors, comorbidities, and baseline imaging parameters were tested using the Student’s *t*-test or one-way between-groups analysis of variance (ANOVA) for continuous variables and Pearson’s chi-square test for categorical variables (or Fisher’s exact tests for cases where a category had fewer than five observations) as appropriate. All analyses were performed in the R project for statistical computing V.3.6.3 (https://www.R-project.org/).

As the PRESERVE and RUN DMC images were analyzed using the same method, individual participant data were pooled and tested again using study site as a covariate. Ordered logistic regression models were performed using the “polr” function in the R package “MASS” (https://www.rdocumentation.org/packages/mass/) to test the association of demographic factors (age, sex, premorbid IQ, and years in education), participant comorbidities (hypertension, hypercholesterolemia, diabetes, and smoking history), and baseline imaging parameters (including MRI hallmarks of SVD, such as lacunar infarcts, cerebral microbleeds, and DTI metrics) with participant category. We used a mixed-effects model with study site as a random factor and other predictors as fixed factors. Selection of predictor variable was conducted using stepwise forward selection of any predictors with *p* value less than 0.05 on univariate analysis and backward selection of predictors that lost this significance in the multivariate model. We also used this method to test the effect of any change in brain volume on WMH regression.

## Results

### Proportion of regressors

**Figure 1.**
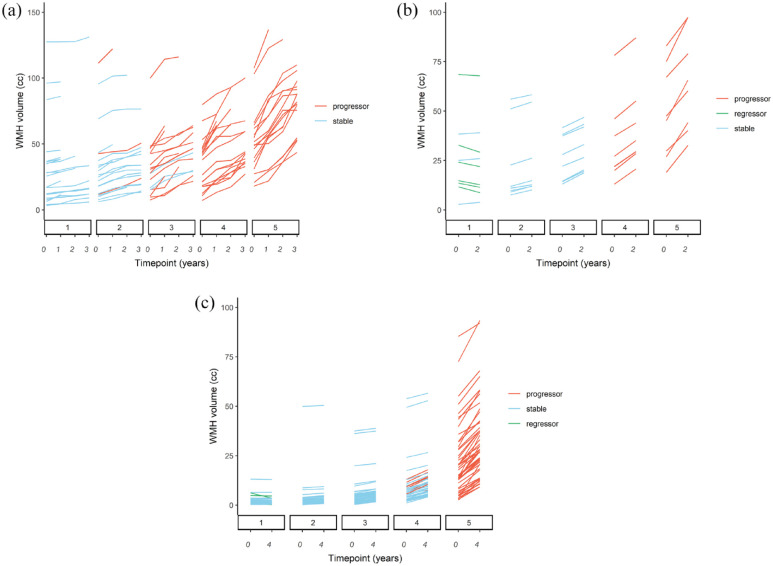
WMH volume change over study duration. Spaghetti plots showing the change in WMH volume in (a) SCANS, (b) PRESERVE, and (c) RUN DMC. Subjects that demonstrated WMH progression tended to have larger baseline WMH volumes while subjects that were categorized as regressors (where present) were exclusively in the bottom quintile of WMH volume of their cohort.

We included 98 participants from SCANS (mean age 69.0 years, 66.3% male, mean baseline WMH volume 37.3 cc) who had four scans over a 3-year period. Of these, no participants showed lesion regression using the definition of a reduction of 0.25 cc. In total, 42 participants from PRESERVE were included (mean age 68.7 years, 65.4% male, mean WMH volume 31.0 cc) who had repeat scans at 2 years. Of these, 6/42 (14.3%) showed lesion regression. We included 276 participants from RUN DMC (mean age 68.1 years, 57.5% male, mean WMH volume 7.75 cc) who had repeat scans at 5 years; of these, 6/276 (2.2%) showed lesion regression. Images from participants who were identified as showing WMH regression were visually inspected and no pairs of images showed discrepancies in image quality/positioning or artifacts that would explain a reduction in WMH volume. Figure 1 shows the WMH volume over time for each of the three cohorts, stratified by quintile of WMH change over the entire study course.

**Figure 2.**
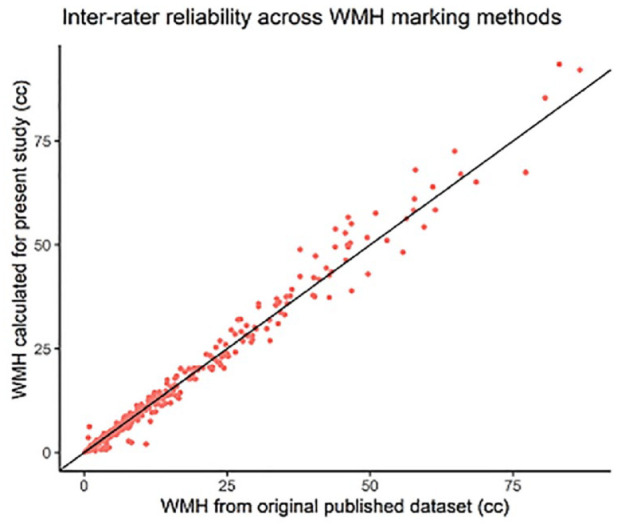
Inter-rater reliability across WMH marking methods. Previously published WMH volumes from RUN DMC versus newly calculated values from this study. The line shows perfect correlation.

There was a very high agreement between the WMH lesion volumes calculated for this analysis and the previously marked and published values in RUN DMC^28^ (intraclass correlation coefficient for both time points 0.992, 95% CI: 0.990–0.993, Figure 2).

### Effects of brain atrophy on proportion of regressors

**Figure 3.**
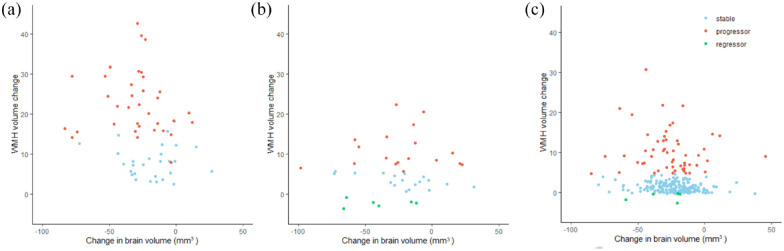
Degree of atrophy versus measured WMH reduction. Scatter plot showing changes in brain volume and WMH measurements over the duration of the study in (a) SCANS, (b) PRESERVE, and (c) RUN DMC.

To test the effect of brain atrophy on lesion measurement, we also calculated WMH volumes normalized by total brain volume at each time point. Using this method, one participant from PRESERVE was reclassified from “regressor” to “stable” leaving 5/42 patients from PRESERVE and 6/276 from RUN DMC as regressors. In a further exploratory analysis, change in brain volume was not associated with WMH regression on univariate analysis (*β*-coefficient 0.178, *p* = 0.12). Figure 3 shows the relationship between change in brain volume and WMH in each study; there were a small number of participants who showed an increase in brain volume which was less than 50 mm^3^ and this is within the expected tolerance of SIENAX.^31^

### Effect of using different thresholds to define regression

We performed a secondary analysis defining “regressors” using a threshold of one standard deviation of the inter-scan WMH change (i.e. the inverse of participants defined as progressors). Using this definition, we found no regressors in any population. Conversely, matching the threshold of WMH growth to define “progressors” to 0.25 cc led to all the participants originally marked as “stable” to be reclassified as “progressors” in all three cohorts, except for one participant in SCANS who showed an WMH increase in less than 0.25 cc between each of the four time points. Differences between participants in binary categories (regressors versus non-regressors) are shown in Supplemental Table 1.

### Factors associated with regression

Table 2 shows the number of regressors, stable, and progressor participants in each study and the differences in demographic, risk factor, and imaging variables between groups. In RUN DMC, regression was associated with lower age. More severe imaging markers of SVD tended to be negatively associated with regression; the WMH regressor group had lower baseline lesion volume, higher brain volume, and MD normalized peak height in RUN DMC, and lower lacune count and higher FA median in both studies. Levene’s test of homogeneity was used to ensure that ANOVA assumptions were valid in each cohort.

**Table 2.** Comparison of demographics, risk factor, and imaging variables at baseline between regressors, stable, and progressors in each cohort, separated by study.

	SCANS cohort (*n* = 98)					PRESERVE trial (*n* = 42)					RUN DMC cohort (*n* = 276)				
	Total population	Regressors	Stable	Progressors	*p* value	Total population	Regressors	Stable	Progressors	*p* value	Total population	Regressors	Stable	Progressors	*p* value
Number	98	0 (0)	41 (41.8)	57 (58.2)	-	42	6 (14.3)	18 (42.9)	18 (42.9)	-	276	6 (2.2)	199 (72.1)	71 (25.7)	**-**
Age	69.0 ± 10.0	n/a	69.4 ± 9.8	68.7 ± 10.2	0.73	67.7 ± 8.4	72.5 ± 5.5	66.9 ± 8.8	66.8 ± 8.8	0.32	68.1 ± 7.4	**61.5** **±** **6.2**	**67.0** **±** **7.3**	**71.8** **±** **7.6**	**<1 × 10** ^−4^
Sex (male)	64 (65.3)	n/a	26 (63.4)	38 (66.7)	0.99	18 (42.9)	3 (50)	9 (50)	6 (33.3)	0.63	158 (57.2)	5 (83.3)	113 (56.9)	40 (56.7)	0.52
NART	99.8 ± 15.3	n/a	99.4 ± 14.9	100.1 ± 15.5	0.83	114.9 ± 9.3	112.7 ± 11.4	114.7 ± 9.1	116.0 ± 8.8	0.77	-	-	-	-	-
Education (years)	11.8 ± 3.3	n/a	11.7 ± 3.0	11.9 ± 3.5	0.81	-	-	-	-	-	11.3 ± 3.3	**10.7** **±** **3.9**	**11.7** **±** **3.3**	**10.4** **±** **3.2**	**0.024**
Hypertension (%)	92.9	n/a	92.7	93.0	1	100	100	100	100	1	78.8	66.7	77.1	84.5	0.33
Diabetes (%)	19.3	n/a	21.9	17.5	0.66	19.0	16.7	16.7	22.2	0.91	15.2	16.7	14.1	18.3	0.72
Hypercholesterolemia (%)	86.8	n/a	87.8	84.2	0.99	78.6	50	83.3	83.3	0.19	50.7	50	44.1	60.6	0.099
Smoking (%)	21.4	n/a	24.3	19.2	0.99	14.3	0	16.7	16.7	0.58	16.7	16.7	16.5	16.9	0.99
Baseline WMH (cc)	37.3 ± 26.2	n/a	**30.1** **±** **27.7**	**42.5** **±** **25.0**	**0.026**	31.0 ± 19.9	27.6 ± 21.6	24.5 ± 15.7	38.7 ± 22.9	0.11	8.17 ± 10.9	**3.0** **±** **2.0**	**4.217** **±** **7.8**	**19.676** **±** **17.0**	**<1 × 10** ^−4^
Brain volume (cc)	1299.4 ± 86.0	n/a	**1313.6** **±** **83.8**	**1289.1** **±** **87.6**	**0.016**	1365.1 ± 127.9	1372.3 ± 121.2	1414.4 ± 122.9	1313.4 ± 134.7	0.065	1064.35 ± 76.5	**1094.9** **±** **94.4**	**1074.9** **±** **73.1**	**1032.2** **±** **83.9**	**<0.002**
Lacunes	4.28	n/a	3.43	4.89	0.20	4.26	**3**	**2.94**	**6**	**0.048**	0.62	**0.33**	**0.39**	**1.26**	**<0.001**
Microbleeds	5.56	n/a	4.78	6.12	0.74	4.06	2	3.82	5	0.73	0.71	0	0.79	0.55	0.73
FA median	0.292 ± 0.03	n/a	**0.301** **±** **0.03**	**0.287** **±** **0.03**	**0.018**	0.335 ± 0.03	**0.345** ± 0.02	**0.344** ± 0.03	**0.322** ± 0.03	**0.046**	0.339 ± 0.03	**0.347** **±** **0.02**	**0.345** **±** **0.03**	**0.321** **±** **0.03**	**<1 × 10** ^−4^
MD normalized peak height (mm^2^/s)	0.0152 ± 0.03	n/a	**0.0165** **±** **0.003**	**0.0144** **±** **0.002**	**0.00049**	0.0134 ± 0.002	0.0143 ± 0.002	0.0140 ± 0.002	0.0125 ± 0.002	0.148	0.134 ± 0.002	**0.0143** **±** **0.002**	**0.0140** **±** **0.002**	**0.0120** **±** **0.002**	**<** **1 × 10**^−4^

Values are given as *n* (%) or *M* ± *SD*. Significant values (*p* < 0.05) are in bold. NART: National Adult Reading Test; WMH: white matter hyperintensities; FA: fractional anisotropy; MD: mean diffusivity.

**Figure 4.**
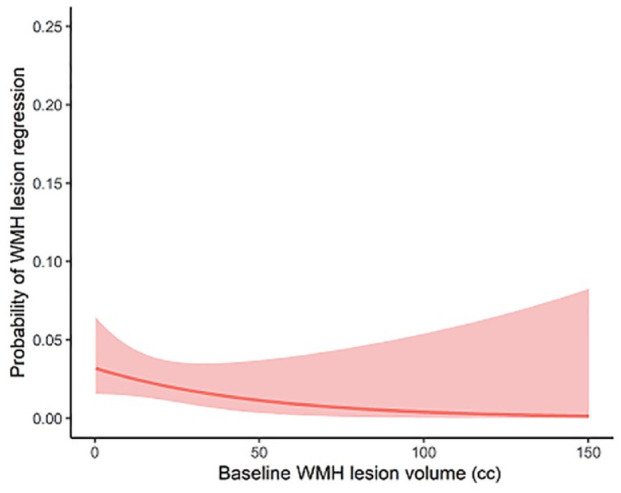
Probability of lesion regression versus baseline WMH volume. Probability of participants showing lesion regression versus baseline WMH volume including 95% confidence interval shaded. WMH regression was less likely with increasing baseline WMH, but there was no clear threshold beyond which there was no lesion regression.

Using pooled individual participant data, significant predictors for WMH regression on univariate analysis were lower age, absence of hypercholesterolemia, lower baseline WMH volume and lacune count, and higher brain volume, FA median, and MD normalized peak height (see Table 3). On multivariate analysis including variables significant on univariate analysis, only baseline WMH volume (OR: 0.36, 95% CI: 0.23–0.56) and baseline FA median (OR: 1.55, 95% CI: 1.07–2.24) were associated with lesion regression. Figure 4 shows how the probability of lesion regression depended on baseline WMH volume across the three cohorts, with regression being associated with lower baseline WMH volume.

**Table 3.** Predictors of WMH regression using ordered logistic regression assessed on both univariate and multivariate analysis.

Predictor variables	Univariate models			Final multivariate model				
	*β*	*SE*	*p* value	*β*	*SE*	*OR*	*t*-statistic	*p* value
Study (RUN DMC vs PRESERVE)	−0.479	0.364	0.18					
Age (years)	**−0.055**	**0.016**	**<0.001**					
Sex (male vs female)	0.259	0.252	0.304					
Hypertension (yes/no)	−0.534	0.341	0.12					
Hypercholesterolemia (yes/no)	**−0.637**	**0.259**	**0.0014**					
Diabetes (yes/no)	−0.295	0.34	0.39					
Smoking (yes/no)	−0.117	0.334	0.73					
Baseline WMH (cc)	**−1.062**	**0.171**	**<1 × 10** ^−4^	**−1.021**	**0.227**	**0.361**	**<1 × 10** ^−6^	**<1 × 10** ^−4^
Brain volume (cc)	**0.520**	**0.131**	**<1 × 10** ^−4^	0.191	0.218	1.85	0.88	0.38
Lacunes (count)	**−0.239**	**0.058**	**<1 × 10** ^−4^					
Microbleeds (count)	−0.017	0.023	0.45					
FA median	**0.736**	**0.140**	**<1 × 10** ^−4^	**0.441**	**0.189**	**1.553**	**2.335**	**0.019**
MD peak height (mm^2^/s)	**0.820**	**0.145**	**<1 × 10** ^−4^	0.054	0.260	1.05	0.209	0.83

β: unstandardized β coefficient; SE: standard error; OR: odds ratio; WMH: white matter hyperintensities; FA: fractional anisotropy; MD: mean diffusivity; Significant values (*p* < 0.05) are in bold.

This multivariate model was re-tested with the participants classified as regressors versus non-regressors (i.e. stable and progressor groups combined). In this model, only baseline brain volume reached statistical significance with minimal contribution to the classification (OR per additional cc of brain volume 1.0007, 95% CI: 1.0002–1.001).

## Discussion

In this study, across three prospective cohorts with symptomatic SVD, we found regression of WMH lesion volume in a small proportion of individuals over follow-up periods of between 2 and 5 years. Using a definition of 0.25 cc to define regression, the percentage showing regression was 0%, 2.2%, and 14.3% in SCANS, RUN DMC, and PRESERVE, respectively. When defining “regressors,” using a threshold of one standard deviation of the inter-scan WMH change, we found no regressors in any population. These values are lower than reported in recent studies.^16–18^

This analysis differs from the most previous studies, in that the three populations included only participants with both neurological symptoms and radiological evidence of SVD, and so our study is likely to have excluded participants with less severe, non-progressive, or incidental pathology. The only factor which consistently predicted regression was a lower severity of SVD defined by other imaging variables, and participants who demonstrated lesion regression had a lower WMH burden at baseline. On multivariate analysis, both WMH volume and white matter ultrastructural integrity, as assessed by FA, were associated with regression. Taken together, these results suggest that reduction in WMH is more likely early in the disease process.

Although imaging data from these cohorts have been published previously,^13,20,32^ the current study applied a novel image analysis method specifically to look for WMH regression and additionally used logistic regression to test for associations with other demographic and radiological factors. Previous analysis of the RUN DMC cohort found lesion regression in 26 participants (9.4%) between 2006 and 2011 and in five participants (1.8%) between 2011 and 2015.^28^ The WMH lesion volumes we calculated, even though obtained by a different method, correlated very highly with previously published values. In our analysis, we only used data from the 2011 and 2015 time points because there was a scanner change between 2006 and 2011, and during this period, our estimate of 2.2% is very similar to the 1.8% previously reported. The much higher proportion of regressors between 2006 and 2011 and 2011 and 2015 might be related to the measurement of WMH on different scanners for the 2006 and 2011 analysis.

In PRESERVE, there were a higher number of regressors. The reason for this is uncertain. Of note, it was a multicentre study with image acquired on multiple scanners, and even in the standard blood pressure treatment arm in PRESERVE, there was a reduction in mean blood pressure reduced by 15.3 mmHg.^13^ It is possible that this led to regression in some patients. Blood pressure lowering has been shown to reduce WMH progression in one clinical trial.^14^

The regression of WMHs observed in previous studies has several possible explanations. It may represent a true biological process, such as the resolution of edema or contraction of lesioned tissue as part of an inflammatory or scarring process (as has been proposed based on the finding of expanded free-water compartment surrounding WMHs^33^). Alternatively, it could also be explained by radiological or technical factors causing apparent regression in some subjects. Such factors could include registration of images between scans, equipment changes between scans, and discrepancies between image quality and slice angle. It might also reflect statistical factors, such as the uncertainty caused by partial volume effects (particularly in periventricular areas), regression to the mean in lesion marking techniques, or differential rates of atrophy affecting the mapping of lesioned tissue at follow-up to baseline space.^19^

Treatment trials in SVD are increasingly using WMHs as an outcome marker, and the SPRINT-MIND trial was the first to show that intensive blood pressure treatment is associated with slower WMH progression.^14^ In this study, the intensive treatment group showed 0.54 cm^3^ less WMH progression than the control group and this is close to the threshold of 0.25 cc that we used to define regressors. These differences are small and by definition difficult to appreciate visually, though in the context of a trial can be clinically important. WMH regression has not been investigated specifically as an outcome in treatment trials.

Strengths of our study included the use of multiple cohorts which included a range of mild-to-moderate-to-severe SVD, at a time interval after stroke where appearances are likely to be stable. The large sample size allowed associations between demographic risk factors/imaging markers and regression to be examined, and the rater being blinded to time point allowed us to minimize any bias in the application of the semi-automated lesion marking technique that we used.

Limitations of our study include the differing intervals between follow-up imaging in each cohort (as we could not rule out regression and then progression or vice versa, particularly in the 5-year intervals between scans in RUN DMC) and the difference in MRI field strength. Previous meta-analysis found that field strength was not significantly associated with the calculation of WMH progression rate^34^ and this might also apply to the assessment of lesion regression; however, further work is required to assess the effect of scanner and field strength on the estimates of lesion progression and regression. There were minor differences in image acquisition between the PRESERVE and RUN DMC studies; however, we harmonized the WMH segmentation as closely as possible by analyzing the raw images using the same lesion marking program/version and on the same PC screen.

We are also limited by the understanding of scan–rescan reliability and though this has been tested in small cohorts with various image analysis techniques,^35,36^ it is still unclear to what extent small fluctuations in WMHs should be expected from imaging across multiple time points. If the noise in WMH calculation is similar to a definitive estimate of natural WMH variation, then the WMH regression we observed in a small number of participants may be pathophysiologically insignificant. Other authors have found that FLAIR signal can decline within WMHs,^37^ and this may indicate that tissue is less severely damaged or less prone to inflammation; our analysis does not account for this phenomenon. It is also possible, particularly in severe cases, that net WMH growth comprises both voxels of de novo WMH in parallel with voxels where WMH revert to normal-appearing white matter. As we focused on whole brain lesion burden, this was not captured in our study. Furthermore, as we identified so few subjects showing regression, it is likely that our study was underpowered to detect other associations with patient and imaging factors.

Our study does not exclude the fact that individual white matter lesions can regress, and this has been clearly demonstrated.^37^ However, although our results may not necessarily generalize to asymptomatic or community populations, they does suggest that in symptomatic SVD, it is unusual for total WMH lesion volume to regress. This is reassuring in the use of WMH as a surrogate endpoint in clinical trials for SVD and suggests that previous power calculations performed without considering regressors are likely to be accurate. Future studies testing interventions in SVD or performing longitudinal analysis of white matter damage in other populations (such as participants with cardiovascular risk factors or cognitive impairment) should consider a similar blind-to-time point analysis method.

## Supplemental Material

sj-docx-1-wso-10.1177_17474930231169132 – Supplemental material for How often does white matter hyperintensity volume regress in cerebral small vessel disease? Click here for additional data file. Supplemental material, sj-docx-1-wso-10.1177_17474930231169132 for How often does white matter hyperintensity volume regress in cerebral small vessel disease? by Robin B Brown, Daniel J Tozer, Marco Egle, Anil M Tuladhar, Frank-Erik de Leeuw and Hugh S Markus in International Journal of Stroke
