# Supplementary material for: How often does white matter hyperintensity volume regress in cerebral small vessel disease?
Source: Int J Stroke. 2023 May 9;18(8):937–47. doi: 10.1177/17474930231169132 (PMC10507994; doi:10.1177/17474930231169132)
Supplement: sj-docx-1-wso-10.1177_17474930231169132 – Supplemental material for How often does white matter hyperintensity volume regress in cerebral small vessel disease? [file sj-docx-1-wso-10.1177_17474930231169132.docx]

**SUPPLEMENTARY MATERIAL.**

Details of the imaging sequences used across the three cohorts were as follows.

**SCANS:** T1-weighted coronal spoiled gradient recalled echo, TR/TE = 11.5/5ms, field of view = 240 $\times$ 240mm^2^, matrix = 256 $\times$ 192, flip angle = 18°, 176 slices at 1.1mm thickness (final resolution 1.1mm isotropic); axial FLAIR, TR/TE/TI = 9000/130/2200ms, field of view = 240 $\times$ 240mm^2^, matrix = 256 $\times$ 192, 28 slices at 5mm thickness (final resolution of 0.47 $\times$ 0.47 $\times$ 5mm). Diffusion tensor images were acquired using an axial single shot spin echo planar sequences (TE= 93.4ms,TR=15600ms) producing 2.5mm^3^ isotropic voxels and whole brain coverage (2.5 mm3; FOV=240 $\times$ 240mm^2^).

**PRESERVE:** Images were acquired on the following MRI scanners: Siemens Prisma, Siemens Magnetom Prismafit, Siemens Verio, Philips Ingenia, Philips Achieva and three Philips Achieva TX. T1-weighted scans were acquired to produce 1mm^3^ isotropic resolution and TR and TE were optimised to ensure T1 weighting/tissue contrast was comparable across sites. FLAIR sequences had identical inversion times and were also TE matched with TR long enough to minimise T1 weighting (representative TR/TE/TI 11000/120/2800ms, final resolution 0.48 $\times$ 0.48 $\times$ 3mm). Diffusion tensor images were acquired using 32 equally spaced non-collinear diffusion gradients at b=1000s/mm^2^ and eight unweighted images at b=0s/mm^2^ (representative TR = 6850ms TE = 75ms; final resolution 2.5mm^3^ isotropic).

**RUN DMC:** T1-weighted 3-dimensional magnetization-prepared rapid gradient-echo image (TR/TE/TI 2250/2.95/850ms, flip angle 15°, final resolution 1.0 mm isotropic) and FLAIR (TR/TE/TI 14240/892200ms, interslice gap 0.5 mm, final resolution 0.5 × 0.5 × 2.5mm). Diffusion tensor images were acquired using 60 diffusion weighted scans at b=900s/mm^2^ and eight unweighted images (TR= 10100ms; TE= 93ms; final resolution 2.5mm^3^ isotropic).

Supplementary Table 1. Baseline patient details of regressor vs non-regressor groups separated by study.

|  | **PRESERVE trial (*n* = 42)** | | | **RUN DMC cohort (*n* = 276)** | | |
| --- | --- | --- | --- | --- | --- | --- |
|  | Regressors  (*n* = 6) | Non-regressors  (*n* = 36) | P value | Regressors  (*n* = 6) | Non-regressors  (*n* = 270) | P value |
| Age | 72.5 | 66.9 | 0.06 | **61.5** | **68.1** | **0.004** |
| Sex (% male) | 50 | 41.6 | 0.66 | 83.3 | 56.9 | 0.39 |
| NART | 112.7 | 115.4 | 0.63 |  |  |  |
|  | | | | | | |
| Hypertension (%) | 100 | 100 | 1 | 66.7 | 79.0 | 0.29 |
| Diabetes (%) | 16.7 | 19.4 | 0.99 | 16.7 | 15.2 | 0.99 |
| Hyperchol-esterolaemia (%) | 50 | 83.3 | 0.32 | 50 | 48.1 | 0.68 |
| Smoking (%) | 0 | 16.7 | 0.15 | 16.7 | 16.5 | 0.99 |
|  | | | | | | |
| Baseline WMH (cc) | 27.568 | 31.562 | 0.69 | **3.04** | **7.96** | **0.00052** |
| Brain volume (cc) | 1372.3 | 1363.9 | 0.88 | 1,094.9 | 1,065.0 | 0.43 |
| Lacunes | 3 | 4.47 | 0.28 | 0.33 | 0.60 | 0.29 |
| CMBs | 2 | 4.43 | 0.35 | **0** | **0.73** | **0.0062** |
| FA (median) | 0.345 | 0.333 | 0.28 | 0.354 | 0.339 | 0.44 |
| MD (peak height) | 0.0143 | 0.0132 | 0.327 | 0.0136 | 0.0135 | 0.87 |
